# Resource and Technical Advance papers: expanding our resources

**DOI:** 10.1172/jci.insight.151152

**Published:** 2021-06-08

**Authors:** Kathleen L. Collins

As research tools and technologies, such as high-throughput sequencing, proteomic, metabolomic, and lipidomic platforms, advance and become more widely available, it has become clear that the databases generated can serve as valuable hypothesis-generating resources. In particular, major databases that describe unique samples or highlight previously undescribed disease-associated pathways are of high priority. While *JCI Insight* has welcomed and published such papers, we have not formally codified our interest. The Editorial Board is pleased to announce the formal expansion of our Technical Advance category to include major informational databases. The category will now be titled Resource and Technical Advance, and papers in this category should have wide-ranging impact, enhance understanding of disease, and spur future research in the field.

Manuscripts reporting major databases are expected to include new insights derived from the database and/or provide a rationale for why the database provides a unique and valuable resource. Papers describing a new technological advance should provide evidence demonstrating interesting biological insight, as well as evidence demonstrating that the new technology is an advance over the status quo. Like all papers published in *JCI Insight*, the tools and databases described are required to be made available to the community at the time of publication. Sequencing studies must be deposited in a MINSEQE-compliant database, and information to access other large data sets, such as proteomics or metabolomics data, must be described in the article. The Editorial Board feels strongly that the utility of these data lies not only in the initial insights made by the authors, but also in the subsequent analysis by other researchers in the field. Collectively, the entire community benefits from the fundamental observations in these important databases.

The Resource and Technical Advance category will continue to include important new research tools and techniques, with an emphasis on research with a demonstrated application toward understanding and/or treatment of disease. In this issue, Cruz-Herranz, Oertel, and colleagues combine in vivo retinal imaging and transcriptional profiling to provide in-depth temporal information on changes in immune cell populations and cell morphology following onset of EAE (1).

We feel that this formal expansion of the category is important for our continued dedication to publishing high-quality work that provides meaningful contributions toward understanding the biology and/or treatment of disease. We look forward to the opportunity to evaluate your work.

## Version 1 06/08/2021

Electronic publication
